# Noncoding RNA-Mediated Epigenetic Regulation in Hepatic Stellate Cells of Liver Fibrosis

**DOI:** 10.3390/ncrna10040044

**Published:** 2024-08-07

**Authors:** Ruoyu Gao, Jingwei Mao

**Affiliations:** Department of Gastroenterology, First Affiliated Hospital of Dalian Medical University, Dalian 116011, China; gaoruoyu@dmu.edu.cn

**Keywords:** hepatic stellate cells, myofibroblasts, liver fibrosis, epigenetics, noncoding RNAs

## Abstract

Liver fibrosis is a significant contributor to liver-related disease mortality on a global scale. Despite this, there remains a dearth of effective therapeutic interventions capable of reversing this condition. Consequently, it is imperative that we gain a comprehensive understanding of the underlying mechanisms driving liver fibrosis. In this regard, the activation of hepatic stellate cells (HSCs) is recognized as a pivotal factor in the development and progression of liver fibrosis. The role of noncoding RNAs (ncRNAs) in epigenetic regulation of HSCs transdifferentiation into myofibroblasts has been established, providing new insights into gene expression changes during HSCs activation. NcRNAs play a crucial role in mediating the epigenetics of HSCs, serving as novel regulators in the pathogenesis of liver fibrosis. As research on epigenetics expands, the connection between ncRNAs involved in HSCs activation and epigenetic mechanisms becomes more evident. These changes in gene regulation have attracted considerable attention from researchers in the field. Furthermore, epigenetics has contributed valuable insights to drug discovery and the identification of therapeutic targets for individuals suffering from liver fibrosis and cirrhosis. As such, this review offers a thorough discussion on the role of ncRNAs in the HSCs activation of liver fibrosis.

## 1. Introduction

Liver fibrosis represents the final outcome of a range of chronic liver diseases, including alcoholic liver disease (ALD), nonalcoholic fatty liver disease (NAFLD), autoimmune hepatitis, virus B/C hepatitis, cholestatic liver diseases, and radiation or parasite infection-induced liver injury [1,2,3]. The activation of hepatic stellate cells (HSCs) is a crucial event in the development of liver fibrosis. This process is initiated by the exposure of the liver to various pathogens, which subsequently results in hepatocellular injury, leading to HSCs activation and the production of collagens that contribute to fibrosis. Although fibrosis is a reparative process, failure to implement effective interventions may result in the progression of fibrosis to irreversible cirrhosis, characterized by the reconstruction of hepatic lobules that destroys the normal liver functions and structures [1].

The activation of HSCs is characterized by a phenotypic alteration [4]. In response to chronic liver injury, HSCs undergo a transformation into myofibroblast-like cells, which become the primary source of extracellular matrix (ECM), migrate from the space of Disse, and have the capacity of proliferation. This process is referred to as myofibroblast transdifferentiation (MTD) [5]. Activation of HSCs into proliferative, fibrogenic myofibroblasts is well established as the central driver of hepatic fibrosis in cases of liver injury. Several signaling pathways play a role in the transdifferentiation of HSCs into an activated, myofibroblast-like phenotype. Important pathways involved in fibrosis progression include transforming growth factor β (TGFβ), platelet-derived growth factor (PDGF), vascular endothelial growth factor (VEGF), and connective tissue growth factor (CTGF) [6]. The activation of HSCs is also facilitated by the Hedgehog (Hh) ligand and its receptor smoothened homolog (SMO) [7], as well as various other regulatory factors including innate immune signaling [8], adipocytokines involving leptin and adiponectin, nuclear receptors such as liver X receptor (LXR) and farnesoid X receptor (FXR) [9], and epigenetic mechanisms. This review focuses on the role of noncoding RNAs (ncRNAs) in mediating epigenetic changes that drive HSCs activation.

**Figure 1:**
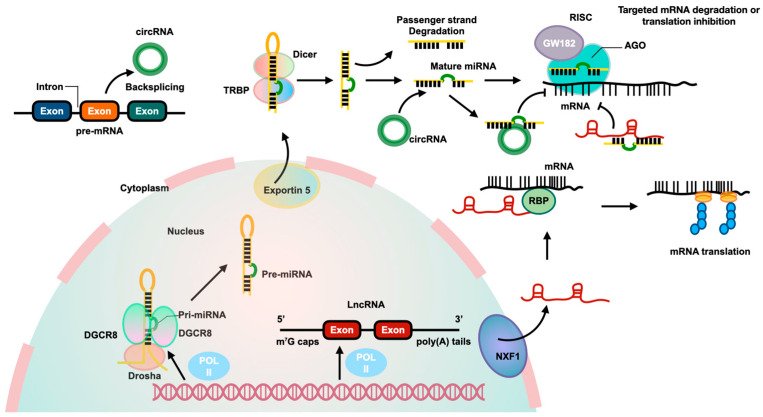
The biological pathways and interactions of three ncRNAs. MiRNAs are produced from pri-miRNAs transcribed by RNA polymerase II from independent genes or introns of protein genes. The pri-miRNAs are processed by a complex involving Drosha and DiGeorge syndrome critical region 8 (DGCR8) to create pre-miRNAs, which are then exported to the cytoplasm by Exportin 5. In the cytoplasm, Dicer cleaves the pre-miRNA to form a miRNA duplex, with one strand being degraded and the other becoming the mature miRNA. The mature miRNA, along with Argonaute2 (AGO2) and glycine-tryptophan repeat-containing protein of 182 KDa (GW182), binds to the 3′-untranslated region (3′-UTR) of targeted mRNA to inhibit its translation. LncRNAs are transcribed by RNA polymerase II and have a structure similar to mRNA. They can be transported from the nucleus to the cytoplasm by nuclear RNA export factor 1 (NXF1). Some lncRNAs act as competitive endogenous RNAs (ceRNAs), binding to miRNAs to affect gene expression. LncRNAs can also interact with RBPs to stabilize and promote mRNA translation. CircRNAs are produced from pre-mRNA through backsplicing and can regulate gene expression by acting as ceRNAs to inhibit miRNA and increase expression of targeted genes.

Epigenetics refers to the persistent modifications of genes, which can be transmitted through cell division in a relatively brief period. These modifications do not entail alterations to the DNA sequence [10]. Increasing evidence indicates that epigenetics plays a significant role in the development and progression of liver diseases [11]. The correlation between epigenetics and liver disease has been established through the utilization of genome-wide association and candidate genes [12]. The augmentation of epigenetic alterations has captured the attention of academics, encompassing ncRNAs, DNA methylation, and histone modifications, which, in conjunction with transcription factors, collectively exert a significant impact on the regulation of specific gene expression. Increasing evidence currently contends that ncRNAs are essential for the control of gene expression. In general, ncRNAs can be divided into microRNAs (miRNAs), long noncoding RNAs (lncRNAs), and circular RNAs (circRNAs). Figure 1 presents the biological pathways and interactions of these three ncRNAs. The integration of ncRNAs with DNA modifications, histone modifications, and transcription can significantly impact the epigenetics of HSCs activation. The aim of this review is to furnish a thorough exposition of the involvement of ncRNAs in the activation of HSCs in the context of liver fibrosis.

## 2. NcRNA-Mediated Epigenetic Regulation in HSCs Activation

### 2.1. Identified MiRNAs in HSCs Activation

MiRNAs, a type of small ncRNAs, are distinguished by their 20–24 nucleotides and play a crucial role in regulating liver functions. The biogenesis of miRNAs is a complex, multi-step process that involves transcription, cleavage, and maturation. Through binding to the 3′-untranslated regions (3′-UTR) of target mRNAs, miRNAs can post-transcriptionally regulate gene expression, leading to the degradation or inhibition of translation of specific genes [13,14]. We focus on elucidating the roles of well-known miRNAs, including the miR-15 family, miR-21, miR-23, and miR-34, in the activation of HSCs and the mechanisms through which they regulate HSCs in the context of liver fibrosis.

The miR-15 family, including miR-15a, miR-15b, miR-16, miR-195, miR-497, and miR-322, demonstrates differential effects on the activation of HSCs, with miR-15a, miR-15b, and miR-16 exhibiting inhibitory effects, while the remaining members exhibit stimulatory effects. MiR-15a can directly target SRY-box transcription factor 9 (SOX9) to decrease its expression; SOX9 downregulation can effectively attenuate HSCs activation [15]. MiR-16 exerts a down-regulatory effect on guanine nucleotide-binding ɑ-subunit 12 (Gɑ₁₂), a protein that is known to facilitate autophagy and promote HSCs activation [16,17,18]. However, miR-195 and miR-497 are positive regulators of the TGF-β1 pathway through binding to the 3′-UTR of Smad7 mRNA, thereby inducing HSCs activation. MiR-195-3p also facilitates HSCs transdifferentiation into myofibroblasts by directly suppressing PTEN (phosphatase and tensin homology deleted on chromosome 10) expression [19,20,21].

MiR-21 has been identified as a potential biomarker for liver cirrhosis [22]. After the Schistosomiasis japonica infection, the Schistoma eggs can produce IL-13 that would stimulate HSCs Smad1/2 phosphorylation and elevate the level of miR-21 to suppress Smad7 expression; finally, the series of changes could cause HSCs activation [23]. In addition to inhibiting Smad7 expression, miR-21 overexpression in activated HSCs can also directly bind to the 3′-UTR of Von Hippel–Lindau (VHL) to suppress its expression, which can further activate NF-κB to promote HSCs to produce cytokines that can aggravate liver inflammation and fibrosis [24]. The Hippo/YAP1 pathway has been shown to involve the upregulation of miR-21, which can activate the Hippo pathway by inhibiting Smad7 expression to promote YAP1 expression [25]. Additionally, miR-21 plays a crucial role in hepatic cells beyond HSCs, as evidenced by its role in inhibiting autophagy in activated HSCs through downregulation of autophagy-related gene 5 (ATG5), leading to enhanced proliferation and reduced apoptosis of myofibroblast-like HSCs by activating the PI3K/AKT/mTOR pathway. In activated HSCs, the downregulation of ATG5 by miR-21 results in the inhibition of autophagy, thereby promoting proliferation and suppressing apoptosis of myofibroblast-like HSCs through the reduction in PTEN and subsequent activation of the PI3K/AKT/mTOR pathway [26].

The miR-23b/27b/24-1 cluster has been demonstrated to mitigate liver fibrosis by directly targeting the mRNAs of profibrotic genes. Additionally, miR-27-3p has the potential to ameliorate liver fibrosis by reducing YAP expression, thereby inhibiting Lysyl oxidase-like 2 (LOXL2) and subsequently suppressing HSCs activation [27]. This effect extends to the downregulation of TGF-β1, Gremlin1, LOX (Lysyl oxidase), Itga2 (integrin 2), and Itga5 (integrin 5) [28]. A previous investigation revealed that miR-23a accumulation in CCL₄-induced liver fibrosis in rats is mediated through the PTEN/PI3K/AKT/mTOR signaling pathway, leading to HSCs activation [29]. Furthermore, the use of prostaglandin E₂ (PEG₂) to induce apoptosis in HSCs can alleviate liver fibrosis by reducing miR-23a-5p levels [30].

As a crucial target of miR-34c, ACSL1 (acyl-CoA synthetase long-chain family member 1) is involved in HSCs activation during liver fibrosis. The downregulation of ASCL1 has been found to be associated with reduced lipid levels, leading to the transdifferentiation of HSCs into myofibroblast-like cells and the promotion of liver fibrosis. Inhibition of miR-34c can restore ACSL1 levels and potentially improve liver fibrosis by restoring lipid metabolism and maintaining HSCs quiescence [31]. The involvement of miR-34 in liver fibrosis remains a topic of debate. One study has demonstrated that miR-34a-5p exerts an anti-fibrotic effect by directly binding to the 3′-UTR of Smad4, thereby inhibiting the TGF-β1/Smad pathway [32].

Up to now, an increasing number of miRNAs are being identified as key regulators in the activation of HSCs through modulation of various signaling pathways, including TGF-β1, Wnt/β-catenin, Hippo, and Hedgehog pathways. A comprehensive overview of these miRNAs is presented in Supplementary Data—Table S1.

### 2.2. LncRNAs Involved in HSCs Activation

LncRNAs are RNA molecules that exceed 200 nucleotides in length and do not undergo translation into proteins. An expanding body of research has elucidated the significant role of lncRNAs in the pathogenesis of liver fibrosis, where they function as competing endogenous RNAs (ceRNAs) by sequestering miRNAs implicated in fibrosis. Additionally, lncRNAs can exert pivotal regulatory functions by interacting with various RNA-binding proteins to enhance the stability of target mRNAs and subsequently augment their protein expression. Recent investigations have identified numerous lncRNAs involved in diverse cellular-level mechanisms governing the activation of HSCs, which are crucial in the progression of liver fibrosis. This section explores the roles of lncRNAs, specifically lncRNA nuclear enriched autosomal transcript 1 (NEAT1) and H19, in regulating HSCs, as well as the impact of other lncRNAs in other liver cell types, including immune cells, on the activation of HSCs.

NEAT1, which is required for paraspeckle formation and integrity, is located in the interchromatin space and plays an important role in retaining RNA. The involvement of NEAT1 in liver fibrosis is evident, as it inhibits miR-139-5p, leading to the upregulation of β-catenin and subsequent promotion of SOX9 expression. This cascade of events initiates the TGF-β1 pathway, ultimately activating HSCs [33]. Moreover, the therapeutic efficacy of miR-122 in mitigating liver fibrosis is compromised by elevated NEAT1 levels, which in turn increase KLF6 expression in HSCs, ultimately resulting in HSCs activation [34]. In a previous study, it was found that NEAT1 targets miR-148-3p and miR-22-3p, leading to a reduction in these miRNAs in fibrotic tissue. This downregulation of miR-148-3p and miR-22-3p by NEAT1 results in increased expression of cytohesin 3, thereby promoting activation of HSCs. Additionally, NEAT1 is shown to decrease the level of miR-29b, which in turn increases Atg9 expression and enhances HSCs activation through the modulation of autophagy [35,36]. Furthermore, NEAT1 directly downregulates miRNA-506, subsequently upregulating the expression of its downstream gene GLI3, a component of the Hedgehog signaling pathway [37].

H19, as a ceRNA, has been demonstrated to play a role in liver fibrosis in cholestatic liver injury by modulating specific targets [38]. H19 promotes the transdifferentiation of HSCs into myofibroblasts via the TGF-β1 pathway, while the downregulation of the antifibrotic miR-148a in liver fibrosis inhibits HSCs activation by targeting ubiquitin-specific protease 4 (USP4). A study has shown that H19 exacerbates liver fibrosis by interacting with miR-148a to induce overexpression of USP4, leading to activation of HSCs by inhibiting degradation of Smad4 or TGF-βRⅠ and enhancing TGF-β signaling [39]. Additionally, hypoxia inducible factor-1ɑ (HIF-1α) induces H19 expression, activating the AMPK pathway to promote degradation of lipid droplets in HSCs, ultimately causing their activation and worsening liver fibrosis [40].

The lncRNA HEIM, originating from monocytes, plays a significant role in promoting activation of HSCs by inducing monocytes to produce increased levels of TGF-β, which then binds to TGF-β receptors on HSCs to stimulate their activation [41]. Conversely, the inhibition of highly upregulated liver cancer (HULC) lncRNA has been shown to effectively mitigate liver fibrosis by preventing hepatocytes from releasing inflammatory molecules upon cell death, thus inhibiting liver inflammation and HSCs activation through suppression of the MAPK pathway [42]. Additionally, the lncRNA HOX transcript antisense intergenic RNA (HOTAIR) has been found to have indirect effects on HSCs activation by directly influencing other hepatic cells. HOTAIR functions as a ceRNA for miR-17a-5p, thereby regulating the transdifferentiation of CD⁴⁺T cells into Th17 cells by enhancing the expression of retinoic acid receptor-related orphan receptor ***γ***t (ROR***γ***t). This upregulation of ROR***γ***t leads to the overexpression of nuclear receptors, resulting in the secretion of IL-17 by Th17 cells to activate HSCs [43]. Supplementary Data—Table S2 offers a comprehensive overview of the expression patterns, functional roles, and regulatory mechanisms of various other identified lncRNAs in liver fibrosis.

### 2.3. CircRNAs Involved in HSCs Activation

CircRNAs have the capacity to function as ceRNAs by sequestering miRNAs, thereby leading to an increase in the levels of miRNA targets and ultimately modulating gene expression at the epigenetic level [44]. Interactions among circRNAs and mRNAs are present in biological processes and contribute to the pathogenesis of multiple diseases, including lymphocyte development regulation, cancer progression, and chronic diseases [45]. Specifically, circRNAs have been implicated in the advancement of chronic liver diseases and hepatocellular carcinoma (HCC), with a notable association with liver fibrosis and HSCs activation. The relationship between circRNAs and fibrosis has been extensively validated through various studies in recent years.

CircMTO1 has been shown to inhibit liver fibrosis by downregulating miR-17-5p, thereby upregulating Smad7 levels to inhibit the TGF-β1 pathway [46]. Additionally, CircMTO1 increases PETN expression by interacting with miR-181-5p to suppress HSCs activation and alleviate hepatic fibrosis [47].

CircRNAs are known to play crucial roles in epigenetics not only within HSCs but also in other types of liver cells involved in hepatic fibrosis. Specifically, circRNAs have been shown to directly stimulate Kupffer cells (KCs) to release various chemokines and inflammatory cytokines, leading to the activation and proliferation of HSCs. One such circRNA, circMcph1, exacerbates liver fibrosis through a similar mechanism by acting as a sponge for miR-370-3p, thereby increasing interleukin-1 receptor-associated kinase 2 (Irak2) expression and intensifying KCs-mediated inflammatory damage and fibrosis through indirect regulation of HSCs [48]. Additional circRNAs implicated in liver fibrosis are detailed in Supplementary Data—Table S3.

**Figure 2:**
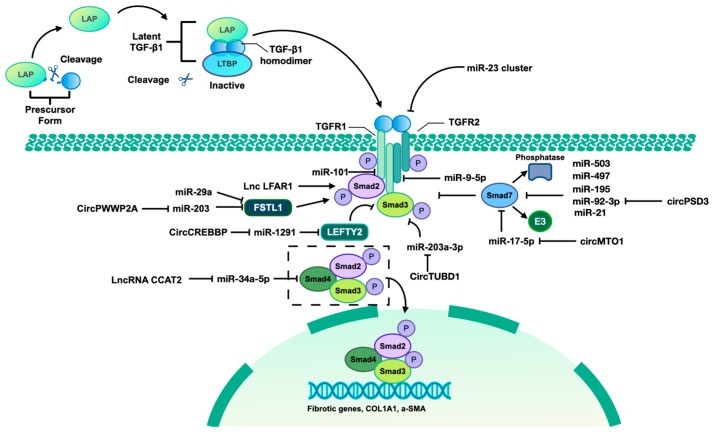
TGF-β1/Smad pathway mediated by identified ncRNAs. The TGF-β signaling pathway involves three isoforms, namely TGF-β1, TGF-β2, and TGF-β3. This discussion will focus specifically on the mechanism of TGF-β1. The precursor form of TGF-β contains a latency-associated peptide (LAP), which can be cleaved to allow binding to the mature TGF-β homodimer. This complex, along with the latent TGF-β-binding protein (LTBP), forms the latent TGF-β/LAP/LTBP complex, maintaining TGF-β in an inactive state unable to interact with TGF-βR I and TGF-βR II. Upon release of the TGF-β homodimer from the complex, it becomes active and can interact with TGF receptor II (TGFR II), leading to TGFR I activation and subsequent phosphorylation of Smad2 and Smad3. The phosphorylated forms of Smad2 and Smad3 form a complex with Smad4, translocate to the nucleus, and bind to the promoter regions of fibrotic genes, thereby inducing their transcription. Additionally, Smad7, acting as an inhibitory Smad, competitively interacts with TGFR I to inhibit the binding between TGFR I and Smad2/3, thereby suppressing the activation of the TGF-β1/Smad pathway. Furthermore, Smad7 recruits E3 ubiquitin ligase and phosphatases to facilitate the degradation and dephosphorylation of Smad2/3. Left–right determination factor 2 (LEFTY2) is a member of the TGF-β protein superfamily, which can inhibit TGF-β1/Smad3 signaling. Follistatin-like 1 (FSTL1) is an inducer of TGF-β1/Smad3 signaling through promoting the Smad3 phosphorylation. Some identified ncRNAs can also regulate the TGF-β/Smad pathway by targeting various components of this signaling cascade.

As indicated previously, it is evident that ncRNAs play a significant role in regulating HSCs epigenetics in the context of fibrosis, primarily through the TGF-β pathway. Figure 2 illustrates the involvement of identified ncRNAs in the activation of HSCs via the TGF-β1/Smad pathway.

**Figure 3:**
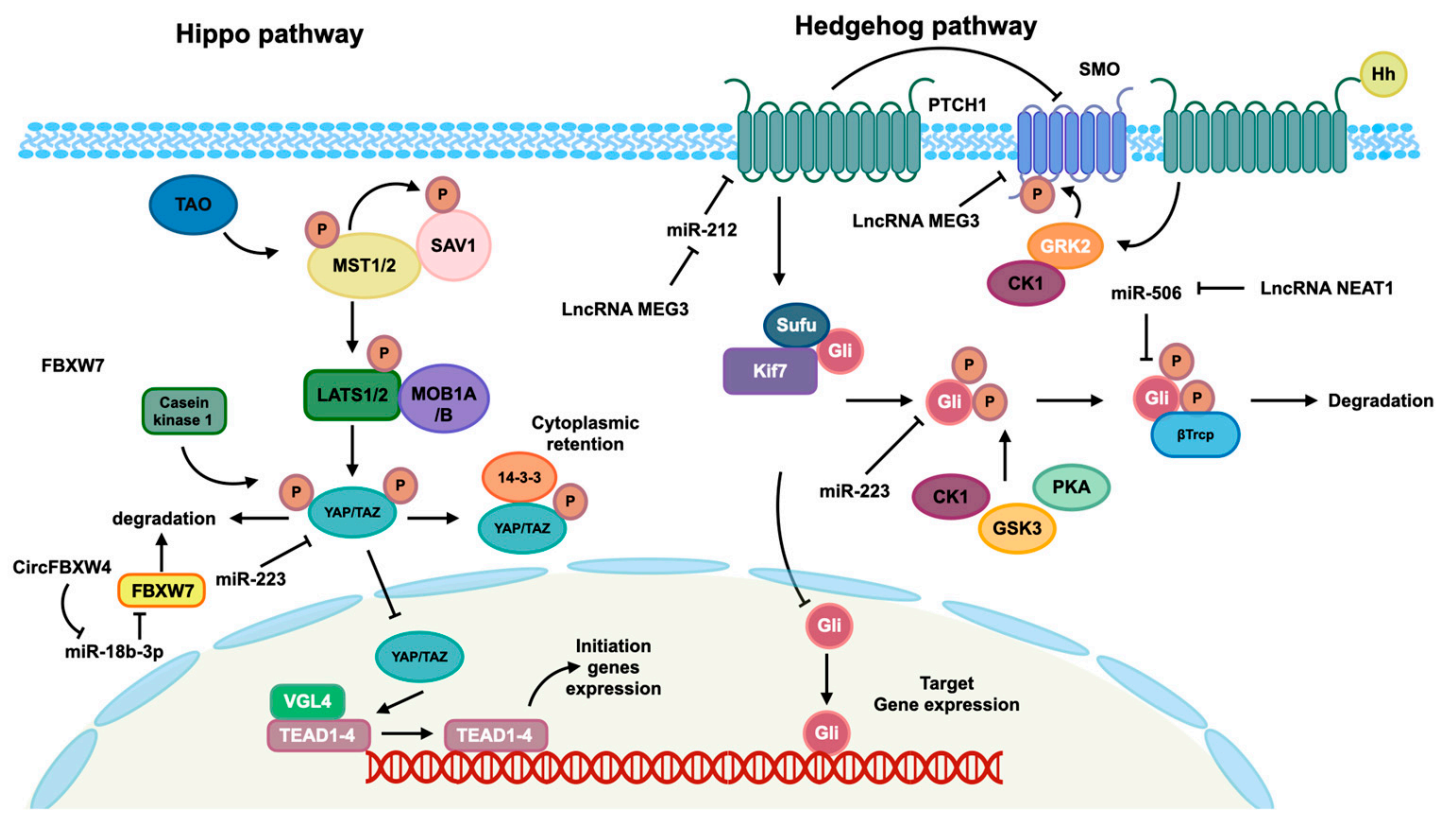
Hippo and Hedgehog pathways mediated by identified ncRNAs. The Hippo pathway is activated by TAO kinase, which subsequently phosphorylates mammalian Ste20-like kinase 1/2 (MST1/2). Phosphorylated MST1/2 then phosphorylates scaffold proteins SAV1 and MOB1A/B, facilitating the recruitment and phosphorylation of the large tumor suppressor 1/2 (LATS1/2). Phosphorylated LATS1/2 facilitates the phosphorylation of transcriptional co-activators Yes-associated protein (YAP) and transcriptional co-activator with PDZ-binding motif (TAZ), leading to their cytoplasmic retention mediated by 14-3-3 and subsequent degradation. In the absence of Hippo pathway activation, the kinase cascades are not initiated, allowing YAP/TAZ co-activators to translocate into the nucleus and interact with the TEAD transcription factor family to modulate gene expression. In the absence of Hedgehog ligands, the twelve-pass transmembrane receptor Ptched (Ptch) is able to inhibit the activity of the seven-pass transmembrane receptor Smoothene (Smo). This inhibition of Smo leads to the inactivation of glioma-associated oncogene transcription factors (Glis) through the formation of a complex with suppressor of Fused (SuFu) and Kif7. This complex then promotes the phosphorylation of Glis by protein kinase (PKA), casein kinaseⅠɑ (CK1ɑ), and glycogen synthase kinase-3β (GSK3β), resulting in the repression of subsequent transcription. When the Hh protein binds to the Ptch receptor, it leads to a reduction in the inhibition of Smo, allowing for the phosphorylation of Smo by CK1 family kinase and GRK2. This phosphorylation event induces the release of Gli from the complex, enabling its translocation into the nucleus to regulate the expression of targeted genes. Additionally, the regulation of these pathways is influenced by ncRNAs, as depicted in this figure.

In addition to the TGF-β pathway, the Hippo and Hedgehog pathways also play a significant role in mediating the activation of HSCs in the context of fibrosis, controlled by ncRNAs. Figure 3 provides insight into the involvement of these two pathways in the regulation of HSCs activation through ncRNAs.

## 3. DNA Modifications Mediated by ncRNAs in HSCs Activation

### 3.1. DNA Methylation in HSCs

**Figure 4:**
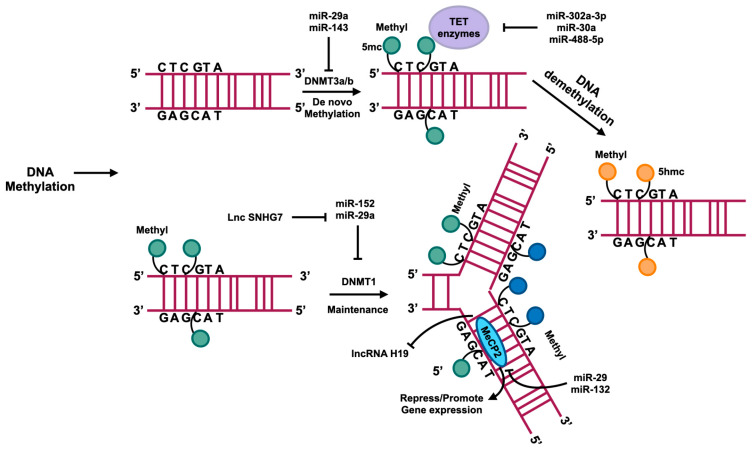
DNA methylation usually occurs in the region enrichment with cytosine–phosphate–guanine (CpG) dinucleotides that are also called CpG islands. DNMTs catalyze the methyl group transference from S-adenyl methionine (SAM) to the fifth carbon of cytosine residue to form 5-methylcytosine (5mc). DNMT3a and DNMT3b catalyze the de novo methylation, namely, add the 5mc to the DNA directly. DNMT1 plays a role in maintaining methylation in DNA replication. A family of TET enzymes can superinduce a hydroxyl group to the 5mc; this process converts the 5mc to 5-hydroxymethylcytosine (5hmc). MecP2 can bind with methylated CpG to suppress or enhance gene expression. The regulation of these processes is influenced by ncRNAs, as depicted in this figure.

DNA methylation is linked to gene silencing, with increased methylation of gene promoters typically leading to reduced or absent gene expression. There are some details about DNA methylation in Figure 4. A recent investigation into the genome-wide epigenetic control of liver fibrosis has unveiled a correlation between hepatic fibrosis and aberrant DNA methylation [49]. Hypermethylation and hypomethylation of genes can promote or inhibit HSCs transdifferentiating into myofibroblasts by inhibiting or inducing the access of transcription factors to DNA for transcription initiation [50,51].

DNA methyltransferases, specifically DNMT1, DNMT3a, and DNMT3b, are responsible for DNA methylation and are associated with de novo methylation processes [52]. Several studies have demonstrated that the above DNA methyltransferases play a role in promoting fibrosis [53,54]. DNMT1 is implicated in DNA methylation maintenance post-replication, with increased expression observed in liver fibrosis and a key role in driving the transformation of HSCs into myofibroblast-like cells [55]. MiR-152 has been shown to downregulate DNMT1 expression, thereby reducing HSCs activation and ameliorating liver fibrosis by inhibiting PTCH1 methylation to enhance its expression and subsequently suppress the Hedgehog pathway [56].

DNMT1 suppresses the expression of lncRNA H19 by enhancing methylation on the promoter region of H19 to downregulate its level, which can further promote the ERK pathway to activate HSCs, although H19 was identified as a fibrotic lncRNA by other research [39,57]. MiR-29a directly targets the mRNA of DNMT1 and DNMT3b to impede the expression of methyltransferases, resulting in PTEN hypomethylation and upregulation, thereby suppressing HSCs activation [58]. Conversely, the lncRNA HOTAIR promotes HSCs activation by inhibiting miR-29a, leading to increased PTEN methylation and reduced PTEN expression [59]. The upregulation of LncRNA Small nucleolar RNA host gene 7 (SNHG7) in activated HSCs leads to its binding with miR-29b, resulting in the enhancement of DNMT3a expression and exacerbation of fibrosis through potential hypermethylation of antifibrotic genes [60]. Elevated levels of DNMT3a are observed in fibrotic liver tissue, and its overexpression is shown to significantly reduce the expression of the lncRNA antisense noncoding RNA in the INK4 locus (ANRIL), possibly by increasing the methylation levels of lncRNA ANRIL to suppress its expression [61]. DNMT3a has been identified as a target of miR-143 in liver tissue and various other organs [62,63]. Additionally, miR-143-3p has been shown to mitigate hepatic fibrosis in autoimmune hepatitis, leading to speculation that miR-143-3p targets DNMT3a to uphold HSCs quiescence [64]. Methyl-CpG binding protein 2 (MeCP2) binds to CpG dinucleotides and serves as a regulator of gene expression, playing a crucial role in HSCs proliferation [65]. MeCP2 plays a role in promoting the conversion of quiescent HSCs into myofibroblasts. Silencing of MeCP2 effectively suppresses this conversion process, while miR-132 inhibits MeCP2 expression, leading to repression of HSCs activation [66]. Additionally, the upregulation of miR-29 reduces MeCP2 expression and suppresses HSCs activation [67]. The lncRNA H19 has its expression mediated by MeCP2. Knockdown of MeCP2 can increase the levels of H19 in HSCs, helping to maintain their quiescent state [68].

### 3.2. DNA Demethylation in HSCs

DNA demethylation is facilitated by the Ten Eleven Translocation (TET) enzyme family, comprising TET1, TET2, and TET3, which are involved in active DNA demethylation linked to the DNA damage response and cell fate determination [69]. The TET family enzymes catalyze the removal of methyl groups by oxidizing 5-methylcytosine to 5-hydroxymethylcytosine [70,71].

While there is no direct evidence suggesting that TET1 plays a role in liver fibrosis, studies have shown its association with fibrosis in other organs. The induction of lung fibrosis by TET1 is mitigated by miR-302a-3p and miR-30a through the downregulation of TET1 expression via binding to the 3′-UTR of its mRNA. This downregulation subsequently inhibits TGF-β1 signaling and prevents mitochondrial fission by suppressing dynamin-related protein 1 expression [72,73]. Conversely, TET3 is implicated in the pathogenesis of liver fibrosis, with its increased expression potentially exacerbating the progression of liver fibrosis. Furthermore, a correlation exists between TET3 and the TGF-β1 pathway. Mir-488-5p inhibits the transdifferentiation of HSCs into myofibroblasts by downregulating TET3 expression, thereby suppressing TGF-β1/Smad signaling [74].

## 4. Histone Modifications Mediated by ncRNAs in HSCs Activation

### 4.1. Histone Acetylation in HSCs

Histone acetylation serves to neutralize the positive charge of histones, thereby diminishing the electrostatic interactions between DNA and histones, resulting in a more relaxed chromatin structure. This relaxed chromatin structure facilitates the access of transcription factors to genes, ultimately influencing the activation or repression of transcription [75].

P300 functions as a co-activator in the TGF-β signaling pathway and possesses acetyltransferase activity, thereby playing a crucial role in the regulation of myofibroblast transformation in HSCs through targeted enhancement of acetylation at histone 3, lysine 18, and lysine 27 (H3K18 and H3K27) [76]. Furthermore, H3K27 at key genes implicated in fibrosis, such as a-SMA, COL1A1, and COL VI, has been linked to the development of fibrosis [77,78]. Previous studies have demonstrated the ability of P300 to induce HSCs activation [79]. Beyond its epigenetic impact on HSCs activation, P300 facilitates the nuclear-cytoplasmic translocation of Smad2/3 and TAZ, ultimately leading to the transcription of fibrotic genes [80].

LncRNA ACTA2-AS1 interacts with P300 in the context of biliary disease-induced fibrosis, serving as a scaffold for the recruitment of the P300/p-ELK1 (a member of the E26 transcription-specific family with the binding sites of P300) complex to the promoter regions of fibrotic genes, thereby promoting their expression [81]. In the setting of liver fibrosis, acetyltransferase 2A (KAT2A) modulates the level of H3K9 acetylation at the promoters of fibrotic genes, potentially through interaction with Smad4, leading to the upregulation of genes associated with HSCs activation, such as fibronectin (FN) and plasminogen activator inhibitor 1 (PAI1) [82].

### 4.2. Histone Deacetylation in HSCs

Histone deacetylases (HDACs) are categorized into four groups: class I, class II, class III (also known as the sirtuin family), and class IV. The regulation of HDACs is closely linked to the fibrosis process. Class I HDACs share similarities with class II HDACs, with the latter being more prevalent in activated HSCs, and inhibiting them can potentially mitigate liver fibrosis. In contrast, class III HDACs, belonging to the Sirtuin family, exhibit distinct protective properties in liver fibrosis compared to the aforementioned classes. NcRNAs have the ability to modulate histone deacetylases, either exacerbating or ameliorating liver fibrosis.

MiR-133a has been found to be downregulated during HSCs activation [83], with HDAC1 and HDAC2 accumulating at enhancer regions of miR-133a to suppress its expression in cardiac fibrosis [84]. However, the validity of this finding in liver fibrosis remains unconfirmed. MiR-455-3p has been shown to decrease in fibrotic HSCs due to hypermethylation of its promoter. Nevertheless, miR-455-3p is capable of downregulating HDAC2 by directly binding to the 3′-UTR of HDAC2 mRNA, thereby inhibiting HSCs proliferation induced by the TGF-β/Smad pathway [85]. Decreasing miR-19b expression has been shown to facilitate the transdifferentiation of HSCs into myofibroblasts [86], while the removal of HDAC3 has been found to alleviate fibrosis through the interaction of miR-19b-3p with the 3′-UTR of HDAC3 in renal fibrosis [87]. This regulatory relationship may also be present in liver fibrosis, as miR-29a has been demonstrated to significantly reduce liver fibrosis by inhibiting HDAC4 activity, leading to increased histone H3 lysine 9 acetylation at the miR-29a promoter and subsequent upregulation of miR-29a expression, thereby exerting an antifibrotic effect [88]. MiR-378 has been shown to indirectly decrease SIRT1 expression by directly stimulating the NF-κB pathway, leading to liver impairment and fibrosis [89]. Additionally, miR-200 has been found to bind to the 3′-UTR of SIRT1, resulting in decreased expression and worsening of liver fibrosis [90].

### 4.3. Histone Methylation and Demethylation

Histone methylation exhibits dual effects on gene transcription, with the ability to either promote or repress gene expression depending on the specific positions at which histone lysine residues are modified. In the context of liver fibrosis, histone methylation on the TGF-β1 promoter, specifically H3K4me1, H3K4me2, and H3K4me3, has been implicated. These modifications have been shown to increase in levels during liver fibrosis, while H3K9me2 and H3K9me3 levels decrease [91]. Additionally, the histone methyltransferase enhancer of zeste homolog 2 (EZH2) is known to mediate H3K27 methylation and has been extensively studied in this context [92]. EZH2 plays a critical role in the activation of HSCs through the methylation of H3K27, resulting in decreased expression of KLF14 and exacerbation of liver fibrosis [93]. Inhibition of EZH2 and overexpression of histone demethylase jumonji domain-containing protein 3 (JMJD3) have been shown to effectively attenuate HSCs activation by reducing H3K27me3 levels on the promoters or gene bodies of profibrotic genes, thereby inhibiting their expression [94]. Additionally, EZH2 is a pivotal regulator of the TGF-β signaling pathway, and downregulation of EZH2 expression has been demonstrated to mitigate liver fibrosis induced by TGF-β [95]. Furthermore, EZH2 plays a role in the Wnt/β-catenin signaling pathway by inhibiting the expression of DKK1, an antagonist of Wnt signaling, thus activating the Wnt/β-catenin pathway and inducing HSCs activation [96].

Certain ncRNAs have the ability to directly regulate the expression of histone methyltransferases, impacting the activation of HSCs. EZH2 is identified as a target of miR-29a, and its overexpression leads to the downregulation of EZH2, thereby mitigating the transformation of HSCs into myofibroblasts [67]. LncRNA H19 binds to EZH2, and this interaction promotes tri-methylation of H3K27, which could induce HSCs activation [97]. LncRNA HOTAIR has the ability to bind directly to miR-148a, thereby increasing the expression of the downstream target DNMT1. This, in turn, leads to an increase in H3K27me3 levels on the promoter of lncRNA MEG3, resulting in decreased expression and subsequent activation of HSCs [98]. Treatment of activated HSCs with the EZH2 inhibitor DZNeP has been shown to alleviate liver fibrosis by downregulating miR-199a-5p expression, a process that may be modulated by EZH2 [99].

## 5. N6-Methyladenosine Mediated by ncRNAs in HSCs Activation

N6-methyladenosine (m⁶A) is a prevalent posttranscriptional modification occurring at the N⁶ position of adenosine within mRNA molecules, playing a significant role in gene expression regulation. Emerging research indicates a correlation between m⁶A modification and HSCs activation. In the context of renal fibrosis, upregulation of METTL3 has been shown to elevate levels of m⁶A in pri-miR-21, leading to the maturation of miR-21-5p. Subsequently, miR-21-5p exacerbates renal fibrosis through the SPRY1/ERK/NF-ΚB signaling pathway [100]. It is plausible to hypothesize that a regulatory relationship exists between m⁶A and pri-miR-21 in the context of liver fibrosis. Acid-sensitive ion channel 1a (ASIC1a) has been shown to enhance METTL3 expression, leading to an increase in m⁶A levels in pri-miR-350. This modification facilitates the binding of pri-miR-350 to DGCR8, a component of the microprocessor complex responsible for maturing pri-miRNAs into mature miRNAs. Furthermore, miR-350 has been found to directly target the downstream gene SPRY2, thereby promoting liver fibrosis [101]. The decreased expression of circIRF2 in liver fibrosis is associated with fibrosis resolution. This decrease is attributed to the m⁶A modification of circIRF2, which enhances the recruitment of the epigenetic reader YTHDF2, leading to the destabilization of circIRF2 and subsequent downregulation of its expression. This downregulation results in the loss of inhibition on miR-29b-1-5p, ultimately leading to the suppression of FOXO3 expression [102].

## 6. The Therapeutic Implications of Noncoding RNAs in Liver Fibrosis

As a non-invasive approach, to test the serum levels of some noncoding RNAs have been biomarkers of liver fibrosis [103]. There are chemical substances that can be implied to attenuate HSCs activation through targeting noncoding RNAs. Sodium–glucose cotransporter inhibitor (SGLT2i) significantly decreases miR-34a-5p expression to inhibit HSCs activation [104]. Dastinib can attenuate liver fibrosis via downregulating miR-17 expression to suppress HSCs activation [105]. Danhongqing formula (DHQ) and salvianolic acid B (Sal B) target lncRNA H19 and lncRNA ROR, respectively, to restrain HSCs activation [106,107]. Exsomes (Exo) derived from different types of cells containing noncoding RNAs, which play an important role in the treatment of liver fibrosis, especially the exosomes derived from mesenchymal stem cells (Msc) [108]. Msc-exos delivering miR-27b-3p and circDIDO1 repress HSCs activation [27,109].

## 7. Conclusions and Perspectives

**Figure 5:**
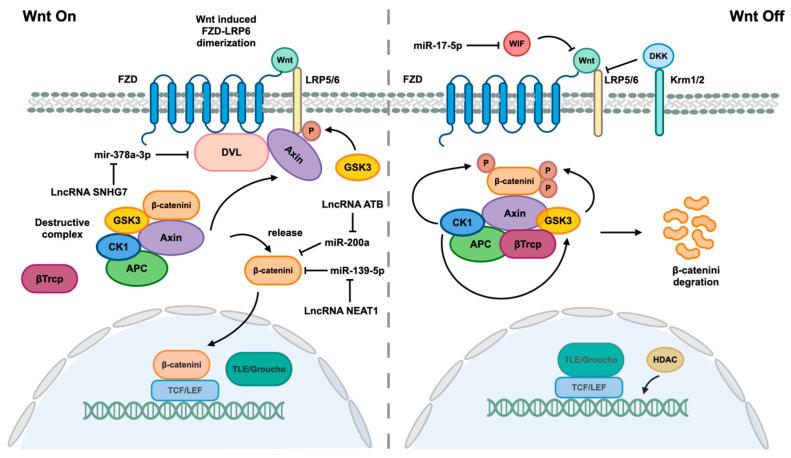
Wnt/β-catenin pathway and associated ncRNAs. When the Wnt signaling pathway is not effectively inhibited by Dickkopf (DKK) or Wnt inhibitory protein (WIF), which interact with lipoprotein receptor-related 5/6 (LRP5/6) to disrupt LRP5/6 and Frizzled (FZD) receptor dimers, Wnt proteins are able to bind to their FZD receptors, leading to dimerization of FZD and LRP5/6 receptors. The formation of FZD/LRP heterodimers induces a conformational change in the receptors, leading to the binding of the cytoplasmic portion of FZD to disheveled (DVL) and phosphorylation of the LRP5/6 tail by GSK3. This phosphorylation event facilitates the recruitment of the scaffold protein Axin. DVL serves as a platform for enhanced interaction between Axin and the LRP5/6 tail. The interaction between Axin, LRP5/6, and DVL disrupts the Destructive Complex (DC), releasing β-catenin. Subsequently, β-catenin translocates into the nucleus, where it displaces corepressor Groucho/transducin with TCF/LEF to form a complex that mediates gene expression. The destruction complex (DC), composed of Axin, adenomatous polyposis (APC), glycogen synthase kinase 3β (GSK3β), and casein kinase 1ɑ (CK1ɑ), functions to maintain β-catenin in an inactive state through phosphorylation by CK1ɑ, facilitating GSK3β-mediated phosphorylation of β-catenin. Subsequent phosphorylation of β-catenin leads to recruitment of β-transducin repeat containing protein (β-Trcp), an E3 ubiquitin ligase that promotes ubiquitination of β-catenin for degradation. The Wnt/β-catenin pathway is also regulated by various ncRNAs.

The activation of HSCs is a complex process that involves various epigenetic modifications, with ncRNAs playing a significant role in driving HSCs to transdifferentiate into myofibroblasts. Additionally, HSCs activation is primarily mediated through four distinct pathways, namely the TGF-β1, Wnt/β-catenin, Hedgehog, and Hippo pathways [110,111]. The figures presented above depict the biological processes and roles of certain identified ncRNAs within the TGF-β1, Hedgehog, and Hippo pathways. Additionally, Figure 5 includes the Wnt/β-catenin pathway and associated ncRNAs that mediate this pathway.

NcRNAs play a role in mediating the aforementioned pathways involved in the activation of HSCs during liver fibrosis. The regulatory mechanism of ncRNAs primarily involves modulating key enzymes responsible for the aforementioned modifications, thereby influencing the expression of genes associated with HSCs activation. Studies on ncRNAs, including miRNAs, lncRNAs, and circRNAs, have demonstrated their ability to form regulatory axes that impact the expression of targeted genes.

Merely focusing on one type of ncRNA is insufficient; it is advisable to investigate the upstream or downstream factors that may influence these RNAs, potentially aiding in therapeutic advancements. Some noncoding RNAs have a dual role in HSCs activation, such as lncRNA H19. Studies think that it has a profibrotic effect, while others hold to the contrary. The mechanisms leading to this phenomenon are currently unclear, so it is worth further study. Furthermore, in addition to the ncRNA-targeted genes axis, an increasing number of studies have revealed that ncRNAs play a role in modulating DNA modifications and post-translational modifications, such as DNA methylation and demethylation, histone acetylation, and deacetylation. The ability of ncRNAs to target multiple genes and signaling pathways suggests a novel approach where manipulating the transcription of one gene may lead to a cascade effect on other genes or pathways, offering potential therapeutic implications.

## Data Availability

All data generated and analyzed during this study are included in this published article.

## Supplementary Materials

The following supporting information can be downloaded at: https://www.mdpi.com/article/10.3390/ncrna10040044/s1. Table S1: Identified miRNAs involved in liver fibrosis title; Table S2: Identified lncRNAs in liver fibrosis; Table S3: Identified circRNAs in liver fibrosis [29,105,109,112,113,114,115,116,117,118,119,120,121,122,123,124,125,126,127,128,129,130,131,132,133,134,135,136,137,138,139,140,141,142,143,144,145,146,147,148,149,150,151,152,153,154,155,156,157,158,159,160,161,162,163,164,165,166,167,168,169,170].
