# Supplementary material for: Noncoding RNA-Mediated Epigenetic Regulation in Hepatic Stellate Cells of Liver Fibrosis
Source: Noncoding RNA. 2024 Aug 7;10(4):44. doi: 10.3390/ncrna10040044 (PMC11357158; doi:10.3390/ncrna10040044)
Supplement: Supplementary file 1 [file ncrna-10-00044-s001.zip › ncrna-3046390-supplementary.pdf]

Table S1. Identified miRNAs involved in liver fibrosis.

MiRNAs	Target genes	Expression	Role	Biological functions	References
**miR-188-5p**	PTEN	Upregulated	Promotion of liver fibrosis	Promoting HSCs activation and proliferation by targeting PTEN/AKT.	Riaz et al., 2021 [112]
**miR-455-3p**	HSF1	Downregulated	Inhibition of liver fibrosis	To inhibit HSCs activation via suppressing HSF1 expression to repress TGF-β1 signaling.	Wei et al., 2019 [113]
**miR-503**	Smad7	Upregulated	Promotion of liver fibrosis	Targeting Smad7 to induce TGF-β1/Smad to activate HSCs	Xie et al., 2021 [114]
**miR-17-5p**	Smad7<br>WIF1	Upregulated	Promotion of liver fibrosis	To activate TGF-β1/Smad and Wnt/β-catenin pathway through suppressing Smad7 and WIFI respectively	Yu et al., 2015 [115]<br>Zhou et al., 2020 [116]
**miR-214-3p**	Sufu	Upregulated	Promotion of liver fibrosis	To induce hedgehog pathway activation to promote HSCs activation	Ma et al., 2018 [117]
**miR-212**	Smad7	Upregulated	Promotion of liver fibrosis	To promote HSCs activation through inhibiting Smad7 expression to initiate TGF-β pathway	Zhu et al., 2018 [118]
**miR-96**	FOXO3<br>Smad7	Upregulated	Promotion of liver fibrosis	To promote HSCs activation via initiating TGF-β1 pathway and enhancing proliferation	Kumar et al., 2023 [119]
**miR-542-3p**	BMP7	Upregulated	Promotion of liver fibrosis	Promoting HSCs activation via inhibiting BMP7 expression to lessen antagonize of TGF-β	Ji et al., 2019 [120]
**miR-708**	TMEM88	Upregulated	Promotion of liver fibrosis	To induce Wnt/β-catenin signaling pathway activation through decreasing TMEM88 expression	Xu et al., 2020 [121]
**miR-122**	P4HA1<br>TGF-β1	Downregulated	Inhibition of liver fibrosis	Impeding HSCs activation through	Li et al., 2013 [122]

				reducing the collagen mature, and inhibiting the signaling of TGF-β1	Nozari et al., 2020 [123]
**miR-618**	Smad4	Downregulated	Inhibition of liver fibrosis	To block HSCs activation by targeting Smad4 to inhibit TGF-β1/Smad pathway.	Sun et al., 2022 [124]
**miR-124-3p**	PI3K	Downregulated	Inhibition of liver fibrosis	To inhibit PI3k/AKT/FOXO3 pathway to induce activated HSCs apoptosis	Li et al., 2021 [125]
**miR-25-3p**	ADAM17<br>FKBP14	Downregulated	Inhibition of liver fibrosis	To restrain HSCs activation through suppressing Notch signaling	Genz et al., 2019 [126]
**miR-3595**	ACSL4	Downregulated	Inhibition of liver fibrosis	Suppressing ACSL4 expression to promote HSCs apaotosis to alleviate fibrosis.	Wu et al., 2018 [127]
**miR-19b-3p**	CCR2	Downregulated	Inhibition of liver fibrosis	To inhibit CCR2 on HSCs expression to suppress monocytes infiltrate into liver to decrease liver inflammation.	Lan et al., 2018 [128]
**miR-137-3p**	PPIC	Downregulated	Inhibition of liver fibrosis	To suppress HSCs activation induced by TGF-β1 through directly decreasing PPIC expression.	Yang et al., 2021 [129]
**miR-130a-3p**	MAPK<br>TGFβR1<br>TGFβR2	Downregulated	Inhibition of liver fibrosis	To induce apoptosis and reduce proliferation and activation of HSCs	Liu et al., 2021 [130]
**miR-671-5p**	Angpt1<br>VWF	Downregulated	Inhibition of liver fibrosis	To inhibit the angiogenic role of HSCs mediated by Angpt1 and VWF	Yang et al., 2022 [131]
**miR-194**	AKT2	Downregulated	Inhibition of liver fibrosis	To inhibit HSCs activation through repressing AKT pathway to further inhibit TGF-β1/Smad pathway activation	Wu et al., 2019 [132]
**miR-345-5p**	HIF-1α	Downregulated	Inhibition of liver fibrosis	To alleviate HSCs activation through decreasing HIF-1α	Wang et al., 2022 [133]

				expression to suppress TGF-β1/Smad signaling	
**miR-193a/b-3p**	TGF-β2<br>CAPRIN1	Downregulated	Inhibition of liver fibrosis	To inhibit TGF-β pathway activation and promote apoptosis to repress HSCs. activation	Ju et al., 2019 [134]
**miR-378**	Wnt-10<br>Gli3	Downregulated	Inhibition of liver fibrosis	To suppress HSCs activation through inhibiting wnt/β-catenin pathway or hedgehog pathway	Zaafan et al., 2022 [105]<br>Hyun et al., 2016 [135]
**miR-489-3p**	JAG1	Downregulated	Inhibition of liver fibrosis	To repress HSCs activation by inhibiting Notch pathway via directly inhibit JAG1, a ligand of Notch	Li et al., 2021 [136]
**miR-155**	TCF4<br>AGTR1	Downregulated	Inhibition of liver fibrosis	To inhibit HSCs activation through suppressing EMT process and ERK1 pathway	Dai et al., 2015 [137]
**miR-139-5p**	PMP22	Downregulated	Inhibition of liver fibrosis	To suppress HSCs activation via inhibiting TGF-β1/Smad pathway by downregulating PMP22 expression	He et al., 2021 [138]
**miR-708**	ZEB1	Downregulated	Inhibition of liver fibrosis	To block HSCs activation via weakening ZEB1 expression to further repress wnt/β-catenin signaling.	Yang et al., 2020 [139]
**miR-148**	Notch2	Downregulated	Inhibition of liver fibrosis	To inhibit HSCs activation via blocking Notch signaling pathway and promote HSCs apoptosis.	Zhou et al., 2022 [140]
**miR-29**	FSTL1	Downregulated	Inhibition of liver fibrosis	To inhibit HSCs activation through decreasing FSTL1 expression to hamper Smad2/3 phosphorylation to inhibit TGF-β1/Smad pathway.	Xu et al., 2020 [141]

Abbreviations: PTEN: phosphatase and tensin homolog; PI3K: phosphatidylinositol-3-hydroxykinase; AKT: protein kinase B; HSF1: heat shock factor 1; TGF-β1: Transform growth factor- beta1; Smad: Small mother against decapentaplegic; WIF1: WNT inhibitory factor 1; Sufu: suppressor-of-fused homolog; FOXO3: Forkhead box protein O3; BMP7: Bone morphogenetic protein 7; TMEM88: Transmembrane protein 88; P4HA1: prolyl 4-hydroxylase subunit alpha-1; PINK1: PTEN-induced putative kinase 1; ADAM17: A Disintegrin and Metalloproteinase 17; FKBP14: FK506 binding protein 14; ACSL4: Long-chain-fatty-acid-CoA ligase 4; CCR2: C-C motif chemokine receptor 2; PPIC: Peptidylprolyl isomerase C; MAPK: mitogen-activated protein kinase; TGFβR: Transform growth factor beta receptor; Angpt1: Angipoietin-1; VWF: Von Willebrand factor; HIF-1α: hypoxia-inducible factor-1alpha; CAPRIN1: cell-cycle-associated protein 1; Gli3: Glioblastoma 3; JAG1: Jagged canonical Notch ligand 1. TCF4: T cell factor 4; AGTR1: Angiotensin II receptor type 1; EMT: Epithelial-mesenchymal transition; PMP22: Peripheral Myelin Protein 22; ZEB: Zinc finger E-box binding homeobox; FSTL1: Follistatin like 1.

Table S2. Identified lncRNAs in liver fibrosis.

LncRNAs	Targets	Expression	Role	Biological functions	Reference
**LncRNA SNHG7**	miR-378a-3p	Upregulated	Promotion of liver fibrosis	Promoting HSCs activation through targeting miR-378a-3p to increase downstream target DVL2 expression, which could lead to wnt/β-catenin pathway initiation.	Yu et al., 2019 [142]
**LncRNA GAS5**	miR-23a	Downregulated	Inhibition of liver fibrosis	To alleviate HSCs activation through impairing the effect of miR-23a to enhance PTEN expression.	Dong et al., 2019 [29]
**LncRNA HOTAIR**	miR-148a-3p	Upregulated	Promotion of liver fibrosis	To stimulate HSCs activation through diminishing the function of miR-148a-3p to enhance S1PR1 expression.	Chen et al., 2023 [143]
**LncRNA ATB**	miR-200a/c	Upregulated	Promotion of liver fibrosis	To activate HSCs via as a ceRNA for miR-200a/c to further elevate β-catenin and ZNF217 expression respectively.	Fu et al., 2017 [144] Zhu et al., 2016 [145]
**LncRNA ANXA2P2**	miR-9	Upregulated	Promotion of liver fibrosis	To initiate HSCs activation through suppressing miR-9 to increase Anxa2 to induce TGF-β1 signaling.	Liao et al., 2022 [146]
**LncRNA CCAT2**	miR-34a-5p	Upregulated	Promotion of liver fibrosis	To aggravate HSCs avtivation through sponging the miR-	Gao et al., 2022 [147]

				34a-5p to enhance Smad4 expression.	
**LncRNA Gpr137b-ps**	miR-200a-3p	Upregulated	Promotion of liver fibrosis	To promote HSCs activation through sponging miR-200a-3p to intensify CXCL14 expression.	Liao et al., 2021 [148]
**lncRNA LFAR1**	Smad2/3 and MTF-1	Upregulated	Promotion of liver fibrosis	To initiate HSCs activation via promoting Smad2/3 phosphorylation to bind with TGFβR Ⅰ and increase MTF-1 expression, both of which can induce TGF-β1/Smad pathway activation.	Zhang et al., 2017 [149] Xuan et al., 2020 [150]
**LncRNA MEG3**	SMO And miR-212	Downregulated	Inhibition of liver fibrosis	To suppress HSCs activation through inhibiting SMO expression or sponging miR-212 to enhance PTCH1 expression to further repress hedgehog pathway.	Yu et al., 2018 [151]
**LncRNA PVT1**	miR-152	Upregulated	Promotion of liver fibrosis	To promote HSCs activation through sponging miR-152 to elevate ATG14 expression to augment HSCs autophagy.	Yu et al., 2020 [152]
**LncRNA MIAT1**	miR-16-5p	Upregulated	Promotion of liver fibrosis	To initiate HSCs activation via lessening the effect of miR-16a-5p to increase TGF-β2 expression.	Ding et al., 2023 [153]
**LncRNA MALAT1**	miR-181a-5p	Upregulated	Promotion of liver fibrosis	To induce HSCs activation via weakening miR-181a-5p to enhance TLR4/NF-κB signaling.	Wang et al., 2021 [154]
**LncRNA HELF**	PIK3R5	Upregulated	Promotion of liver fibrosis	To initiate HSCs activation through interacting with RNA binding protein PTBP1 to keep PIK3R5 stable,	Han et al., 2023 [155]

**lncRNA ROR**	miR-145	Upregulated	Promotion of liver fibrosis	which can activate downstream AKT pathway. To aggravate HSCs activation via sponging miR-145 to increase ZEB2 expression that can activate HERC5 to further decrease p53 expression by promoting its ISGylation and degradation.	Shen et al., 2022 [156]

Abbreviations: SNHG7: host gene 7; DVL2: dishevelled segment polarity protein 2; GAS5: growth arrest-specific transcript 5; HOTAIR: HOX transcript antisense intergenic RNA; S1PR1: Sphingosine 1-phosphate receptor 1; ATB: Activated by TGF-β; Anxa2: Annexins A2; CCAT2: Colon cancer associated transcript 2; Gpr137b-ps: G protein-coupled receptor 137B; CXCL14: C-X-C motif chemokine ligand 14; LFAR1: liver fibrosis-associated lncRNA1; MTF-1: Metallic transcription factor-1; MEG3: maternally expressed gene 3; SMO: Smoothened; PVT1: plasmacytoma variant translocation 1; MIAT: myocardial infarction associated transcript; ATG14: autophagy-related gene 14; MALAT1: Metastasis associated lung adenocarcinoma transcript 1; TLR4: Toll like receptor 4; NF-κB: nuclear factor –κB; HELF: High expressed in liver fibrosis; PIK3R5: phosphoinositide 3- kinase regulatory subunit 5; PTBP1: polypyrimidine tract-binding protein 1; ROR: regulator of reprogramming; ZEB2: Zinc finger E-box binding homeobox 2; HERC5: HECT and RLD domain containing E3 ubiquitin protein ligase 5.

Table S3. Identified circRNAs in liver fibrosis.

CircRNA	Targets	Expression	Role	Biological functions	Reference
**Circ DIDO1**	miR-141-3p	Downregulated	Inhibition of liver fibrosis	To inhibit HSCs activation through sponging miR-141-3p expression to increase the level of PTEN.	Ma et al., 2022 [109]
**Circ PSD3**	miR-92b-3p	Downregulated	Inhibition of liver fibrosis	To suppress HSCs activation via sponging miR-92b-3p to elevate Smad7 to repress TGF-β/Smad pathway.	Bu et al., 2021 [157]
**Circ FBXW4**	miR-18b-3p	Downregulated	Inhibition of liver fibrosis	To mitigate HSCs activation via binding to miR-18b-3p to make FBXW7 overexpression, which could promote YAP-1 and Notch degradation.	Chen et al., 2020 [158]
**Circ CREBBP**	miR-1291	Downregulated	Inhibition of liver fibrosis	To attenuate HSCs activation through	Yang et al., 2020 [159]

				repressing miR-1291 effect to increase the expression of LEFTY2 that can inhibit Smad3 phosphorylation to suppress TGF-β1 signaling.	
**Circ PWWP2A**	miR-203/miR-223	Upregulated	Promotion of liver fibrosis	To induce HSCs activation by sponging miR-203 and miR-223 to enhance FSTL1 and TLR4 expression respectively to induce TGF-β1 and NF-κB pathways.	Liu et al., 2019 [160]
**Circ TUBD1**	miR-203a-3p	Upregulated	Promotion of liver fibrosis	To promote HSCs activation through repressing the function of miR-203a-3p to enhance its downstream target Smad3 expression.	Niu et al., 2022 [161]
**Circ RSF1**	miR-146a-5p	Upregulated	Promotion of liver fibrosis	To induce HSCs activation via impeding miR-146a-5p expression to elevate the level of RAC1 which can mediate the HSCs transform to the fibrotic phenotype.	Chen et al., 2020 [162]
**Circ SCAR**	ATP5B	Downregulated	Inhibition of liver fibrosis	To inhibit HSCs activation by directly binding to ATP5B to repress the ROS production.	Zhao et al., 2020 [163]
**Circ Ube2K**	miR-149-5p	Downregulated	Inhibition of liver fibrosis	To suppress HSCs activation through binding with miR-149-5p to delete its function of suppressing TGF-β2 expression.	Zhu et al., 2021 [164]
**Circ_0071410**	miR-9-5p	Upregulated	Promotion of liver fibrosis	To promote HSCs activation via repressing the antifibrotic function of miR-9-5p to restore the fibrotic targets.	Chen et al., 2017 [165]

**Circ ASPH**	miR-139-5p	Upregulated	Promotion of liver fibrosis	To initiate HSCs activation via suppressing miR-139-5p effect to increase Notch1 expression.	Meng et al., 2023 [166]
**Circ608**	miR-222	Downregulated	Inhibition of liver fibrosis	To repress HSCs activation through inhibiting miR-222 the effect to upregulate PINK1 expression which can mediate HSCs mitophagy to alleviate them activation.	Xu et al., 2022 [167]
**Circ_0000623**	miR-351-5p	Downregulated	Inhibition of liver fibrosis	To mitigate HSCs activation through restoring the autophagy flux by sponging the miR-351-5p function to enhance TFEB expression.	Du et al., 2023 [168]
**Circ_0070963**	miR-223-3p	Downregulated	Inhibition of liver fibrosis	To inhibit HSCs activation via sponging miR-223-3p to enhance LEMD3 expression that can antagonize TGF-β1Smad signaling.	Ji et al., 2020 [169]
**Circ cVIM**	miR-9-5p/miR-122-5p	Upregulated	Promotion of liver fibrosis	To attenuate HSCs activation by protecting TGFβR1, TGFβR2 and KLF6 against Downregulated by miR-9-5p and miR-122-5p.	Zhou et al., 2024 [170]

Abbreviations: DIDO1: Death-Inducer Obliterator 1; PSD3: Pleckstrin and Sec7 domain-containing 3; FBXW: F box and WD 40 domain containing protein; Yap1: Yes associated protein 1;; CREBP: CREB-binding protein; PWWP2A: PWWP domain containing 2A; TUBD1: Tubulin delta 1; RSF1: Remodeling spacing factor 1; RAC1: Ras-related c3 botulinum toxin substrate 1; ATP5B: ATP synthase F_1 subunit beta; SCAR: steatohepatitis-associated circRNA ATP5B regulato; ROS: reactive oxygen species; UBE2K: Ubiquitin-conjugating enzyme E2K; ASPH: Aspartate β-hydroxylase; TFEB: Transcription factor EB; LEMD3: LEM domain-containing protein 3; cVIM: circulation vimentin.
